# Corneal biomechanical changes in eyes with small incision lenticule extraction and laser assisted in situ keratomileusis

**DOI:** 10.1186/s12886-016-0304-3

**Published:** 2016-07-26

**Authors:** Ihab Mohamed Osman, Hany Ahmed Helaly, Moones Abdalla, Mohsen Abou Shousha

**Affiliations:** 1 Department of Ophthalmology, Faculty of Medicine, Alexandria University, Alexandria, Egypt; 2 Roayah Vision Correction Center, Alexandria, Egypt

**Keywords:** SMILE, Corvis ST, Biomechanics, LASIK

## Abstract

**Background:**

Evaluating the corneal biomechanical changes using the Ocular Response Analyzer and the Corvis ST in eyes with incision lenticule extraction (SMILE) and laser assisted in situ keratomileusis (LASIK).

**Methods:**

This is a retrospective study that included 50 eyes equally divided into two groups. The first group included eyes that underwent SMILE procedure using VisuMax® 500 kHz laser system (Carl Zeiss Meditec, Jena, Germany) and the second group included eyes that underwent LASIK procedure using the EX500 Allegretto excimer laser platform (Wavelight GmbH, Erlangen, Germany). The Ocular Response Analyzer (ORA) and the Corvis ST (CST) measured the corneal biomechanical changes before and after the procedures.

**Results:**

The ORA showed significant decrease of corneal hysteresis (CH) and corneal resistance factor (CRF) in both groups postoperatively. The percentage of change of CH and CRF were found to be significantly higher in group II. There was no significant difference in the IOP with the ORA and the CST pre and postoperatively in either group. Using CST, the deformation amplitude and HC peak distances increased significantly in both groups. It was also noted that the mean percentage of change of the deformation amplitude was nearly five times higher in group II than group I.

**Conclusion:**

Both LASIK and SMILE substantially decreased the corneal biomechanical properties with greater reduction in the LASIK group.

## Background

The cornea has been the center of concern for ophthalmologists in the past two decades because of the great advances in corneal refractive procedures. However, the fear of developing ectasia, with such simple procedures, drew the attention of investigators to the importance of understanding the concept of corneal biomechanics. The cornea acts as a viscoelastic material having elements of both viscosity and elasticity. This means that the stress–strain relationship of the cornea is nonlinear, during both the loading and unloading phases [1]. A second important concept is that the response to an applied force has a time-dependent component with a faster strain rate producing a stiffer corneal response [2, 3]. The corneal biomechanics may also provide the explanation of the variable refractive outcomes to patients with otherwise similar demographics undergoing nearly identical surgical procedures [4].

Prior to 2005, the only method of assessing the corneal biomechanics was only in cadaveric eyes. The Ocular Response Analyzer (ORA; Reichert Corporation; Depew, USA) was developed as a noncontact tonometer negating the influence of corneal biomechanics and corneal thickness on intraocular pressure measurements [5]. A 25-millisecond air pulse generated by the machine applies pressure to the cornea to produce an initial applanation followed by progressive inward concavity of the cornea. This initial applanation triggers the cessation of the air puff but because of the inertia of the air puff piston, the air puff continues for fractions of a second causing the inward corneal concavity. At this point the cornea unwinds to go into a second applanation phase until reaching its original state. This Corneal deformation is recorded via an electro-optical infrared (IR) detection system [5–8].

The Corvis ST (CST) (Corneal Visualization Scheimpflug Technology, Oculus, Wetzlar, Germany) was recently introduced in 2010 as a new tool for measuring IOP and corneal biomechanics. This device captures sequential horizontal Scheimpflug images using a high-speed camera during corneal deformation in response to a metered air puff [9]. The machine takes more than 4300 frames per second with a fixed maximal internal pump pressure of 25 kpa. The Corvis ST (CST) camera has a blue light-emitting diode (455 nm, ultraviolet free) covering 8.5 mm of a single horizontal slit. The machine acquires 140 digital frames with 576 possible measuring points in 30 ms. The recording starts with the cornea at the natural convex shape. The machine captures the time and length of the applanated cornea during its inward and outward movement in response to the air puff. The point of maximum corneal concavity is automatically captured and generates several corneal biomechanical parameters [10, 11].

The introduction of femtosecond laser in ophthalmology opened a new era of refractive procedures. The method of flap creation weather by microkeratome or by femtosecond laser had an effect on corneal biomechanics as stated in several studies [12–14]. With recent advances, small incision lenticule extraction (SMILE) procedure now allows for removal of corneal stromal lenticules of predetermined thickness without the need of creating a flap [15, 16]. How this flapless procedure affect corneal biomechanics is not fully investigated yet. This would be considered the first in vivo comparative case series evaluating corneal biomechanics using two different devices namely Ocular Response Analyzer (ORA) and the Corvis ST (CST) in cases of femto SMILE versus the microkeratome assisted LASIK procedures.

## Methods

A retrospective clinical study included 50 eyes. Fifty eyes that were enrolled in the study were equally divided into two groups; group I underwent femto SMILE procedure and group II underwent wavefront optimized LASIK procedure. Records of patients were reviewed. All patients had a thorough preoperative assessment. Dry eye testing including Schirmer and tear breakup time tests were done to exclude cases with dry eye. Patients were excluded from the study if they did not have at least 1 month follow-up with all parameters recorded. Informed consents for retrospective data analysis were obtained from the candidates and the study was approved by the local ethics committee at the Faculty of Medicine, Alexandria University, Egypt. Tenets of Declaration of Helsinki were followed.

VisuMax® 500 kHz laser system (Carl Zeiss Meditec, Jena, Germany) platform was used for the SMILE surgeries. The spot distance was 3 μm for lamellar cuts and 2 μm for side cuts. The spot energy was set to 130 nanojoules in all patients. The minimum lenticule side cut thickness was set to 10 μm. Lenticule side cut angle was 130°, and the incision side cut angle was 70° and the optical zone was 6.5 mm as SMILE is not performed in patients with a mesopic pupillary diameter greater than 6.5 mm. A small-sized cone was used in all patients; cap diameter was set to 7.5 mm. A cap of 90 μm was used in all patients.

Wavefront optimized LASIK procedures were done using the myopic astigmatism algorithm of the EX500 Allegretto excimer laser platform (WaveLight GmbH, Erlangen, Germany). This algorithm uses a 6.50 mm optical zone with a1.25 mm blend zone. All flaps were created using the Sub Bowman keratectomy SBK automated disposable microkeratome with a 90 μm head (Moria, Antony, France).

The postoperative medication regimen consisted of topical steroid and antibiotic drops four times daily for 1 week. The corneal biomechanics were assessed before surgery and 1 month after surgery using both the Ocular response analyzer (ORA) and the Corvis ST (CST). A qualified technician experienced in the study protocols obtained the ORA waveforms and the CST measurements. The ORA and CST procedure were well tolerated by all the patients included. Only good quality ORA and CST measurements were included in the statistical analyses. For each eye, the average of three good quality measurements was recorded.

Four parameters are produced by the ORA namely; corneal hysteresis (CH), corneal resistance factor (CRF), Goldman-correlated IOP (IOPg) and corneal-compensated IOP (IOPcc). These parameters are based on the two pressure measurements at applanation, P1 in the inward direction and P2 in the outward direction. CH is a measure of viscous damping in the corneal tissue, or the energy absorption capability of the cornea. The CRF parameter is a measure of the cumulative effects of both the viscous damping and elastic resistance of the cornea producing the maximum correlation with the central corneal thickness and the IOPcc was designed to be similar before and after refractive surgery [7, 8]. While the CST was used to measure the A1 time, HC time, A2 time, A1 and A2 lengths, HC radius of curvature, peak distances and deformation amplitude (Table 1).

**Table 1:** The list of abbreviations used and their relevance

Abbreviation	Definition
CH CRF IOPcc IOP CST A1 time HC time A2 time A1 length A2 length HC peak distance (HCPD) Deformation amplitude (DA) HC radius	Corneal hysteresis Corneal resistance factor Intraocular pressure corneal thickness compensated Is the intraocular measurement based on A1 time Is the time from starting until first corneal inward applanation Time from starting until highest corneal concavity is reached Time from starting until second corneal outward applanation Cord length of applanated cornea during A1 Cord length of applanated cornea during A2 Distance between corneal peaks at point of highest concavity (HC) Maximum inward movement of corneal apex at point of HC Radius of curvature of the corneal concavity at point of HC

Statistical analysis was performed using Statistical Package for Social Sciences SAS software (version 9.1, SAS Institute, Inc.). Arithmetic mean and standard deviations were calculated for both groups. Sample size was calculated based on a previous study, by using Med Calc statistical software, assuming area under ROC curve to be 0.80, an alpha of 0.05 and power of study 80.0 %. A minimum sample size required for this study was 25 patients. The changes in CH, CRF, IOPcc, IOP Corvis, A1time, HC time, A2 time, A1 length, A2 length, HC radius, HC peak distance and deflection amplitude that were defined as the differences between the preoperative and postoperative parameter then the percentage of change of these parameters were calculated for comparison. To check for normal distribution, the Kolmogorov-Smirnov test was applied. Comparisons of the means of normally distributed data were performed with the t tests for paired samples (for pre- and post-operative comparison) and the t tests for two samples (for comparison between the two groups). A *P* value less than 0.05 was considered statistically significant.

## Results

The study included 50 eyes of normal healthy individuals seeking refractive surgery. Table 2 shows the preoperative characteristics of both groups with no significant statistical difference between them as regards age, sex distribution, preoperative sphere, cylinder, SE, CCT, K1, and K2.

**Table 2:** The mean values and range of age, sex, sphere, cylinder, spherical equivalent (SE), preoperative central corneal thickness (CCT) as well as K1 and K2 values in both groups

	Group I	Group II	*P* value
Age (years)			
Mean ± SD Range	26.28 ± 3.41 21–33	26.88 ± 3.99 22–35	0.285
Gender			
Male Female	12 13	11 14	0.23
Sphere (diopters)			
Mean ± SD Range	−4.90 ± 1.26 −3 to −7.75	−4.59 ± 1.40 −3 to −7.5	0.208
Cylinder (diopters)			
Mean ± SD Range	−1.05 ± 0.52 0 to −1.75	−1.14 ± 0.35 −0.5 to −1.75	0.236
SE (diopters)			
Mean ± SD Range	−5.43 ± 1.17 −3.625 to −8.5	−5.16 ± 1.42 −3.5 to −7.75	0.238
Preop. CCT (microns)			
Mean ± SD Range	532.84 ± 16.37 512–572	527.96 ± 16.21 510–563	0.147
K1 (diopters)			
Mean ± SD Range	44.84 ± 1.04 43–47	44.31 ± 1.39 42.1–47	0.066
K2 (diopters)			
Mean ± SD Range	43.59 ± 1.09 41.8–45.6	42.98 ± 1.48 40–45.7	0.061
Post op SE			
Mean ± SD Range	−0.51 ± 1.02 +0.75 to −1.25	−0.6 ± 0.9 +0.4 to −1.5	0.149
Post OP. CCT			
Mean ± SD Range	486.81 ± 18.32 468–514	479.46 ± 15.71 461–520	0.138

**Fig. 1:**
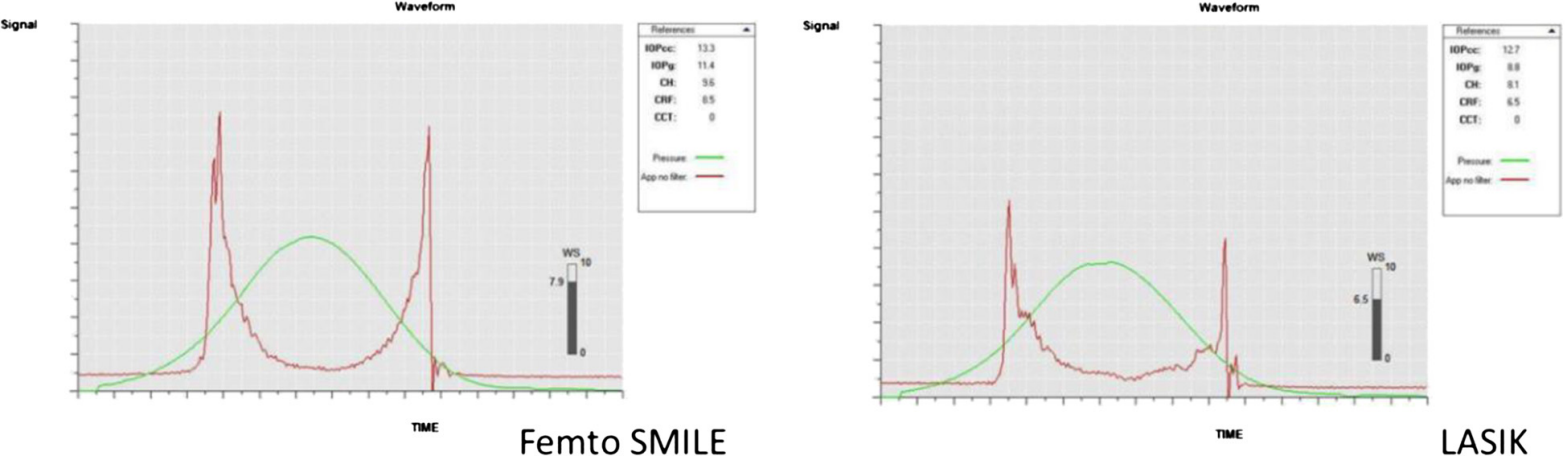
showing higher corneal biomechanical values namely CH and CRF in Group I (on the left) versus Group II (on the right). Note the higher P1 and P2 waves in Group I

Table 3 shows the preoperative and postoperative corneal biomechanics measured by the ORA and CST for both groups thus representing the effect of the surgical procedure on these corneal biomechanics in each group. The ORA showed significant decrease of CH and CRF in both groups postoperatively as compared to the preoperative values. However, there was no significant difference between the preoperative and postoperative IOPcc in both groups (*p* = 0 .313 and 0.117 respectively) (Fig. 1).

**Table 3:** Showing the corneal biomechanical parameters in both groups preoperatively and postoperatively

	GROUP I		GROUP II	
	Preop.	Postop.	Preop.	Postop.
CH				
Range Mean ± SD	7.9–15.1 12.03 ± 1.76	6.9–13.7 9.99 ± 1.76	7.3–11.66 11.59 ± 1.86	4.8–11.1 8.46 ± 1.76
*P* value	0.001*		0.002*	
CRF				
Range Mean ± SD	7.5–15 11.42 ± 1.68	6.5–12 9.43 ± 1.55	6.8–14.2 11.00 ± 1.89	3.69–12.4 7.45 ± 2.39
*P* value	0.003*		0.001*	
ORA IOPcc				
Range Mean ± SD	9.6–21 14.89 ± 3.15	10.2–19.89 14.47 ± 2.88	9.6–19.5 15.59 ± 3.23	8.9–18.9 14.54 ± 2.96
*P* value	0.3139		0.1172	
CST IOP				
Range Mean ± SD	9–21 14.89 ± 2.84	8.9–20 13.20 ± 2.86	8.6–20 15.07 ± 3.02	7.9–18.3 13.54 ± 2.96
*P* value	0.20		0.382	
A1 time				
Range Mean ± SD	8–9.1 8.40 ± 0.36	7.4–9.1 8.23 ± 0.37	7.4–9.2 8.40 ± 0.39	6.9–8.9 7.89 ± 0.44
*P* value	0.069		0.001*	
A2 time				
Range Mean ± SD	21.2–25.9 23.64 ± 1.03	20.2–24.2 22.03 ± 1.11	21.3–25.7 23.42 ± 1.20	16.8–23 20.28 ± 1.87
*P* value	0.001*		0.001*	
A1 length				
Range Mean ± SD	1.5–2.5 2.10 ± 0.22	1.56–2.23 1.90 ± 0.20	1.61–2.49 2.10 ± 0.23	1.46–2.36 1.93 ± 0.23
*P* value	0.0006*		0.0063*	
A2 length				
Range Mean ± SD	1.42–2.3 1.90 ± 0.20	1.39–2.1 1.75 ± 0.20	1.49–2.28 1.90 ± 0.24	1.4–2.1 1.81 ± 0.21
*P* value	0.0061*		0.0622	
HC time				
Range Mean ± SD	16.5–20.1 18.39 ± 0.92	14.9–18.9 16.32 ± 1.10	16.4–19.2 17.74 ± 0.71	12.5–17.4 14.40 ± 1.27
*P* value	0.001*		0.001*	
HC radius				
Range Mean ± SD	6.89–12.99 7.99 ± 1.35	5.2–10.73 6.91 ± 1.25	6.89–11.03 7.69 ± 1.14	5.7–10.23 7.00 ± 1.06
*P* value	0.0025*		0.0152*	
HC Peak Distance				
Range Mean ± SD	3.1–5.3 4.09 ± 0.69	5.56–5.97 4.72 ± 0.71	2.99–4.9 3.81 ± 0.49	3.56–6.2 4.90 ± 0.67
*P* value	0.0013*		0.001*	
Deformation Amplitude				
Range Mean ± SD	0.9–1.23 1.05 ± 0.08	0.94–1.28 1.10 ± 0.08	0.92–1.23 1.02 ± 0.10	1.17–1.45 1.26 ± 0.07
*P* value	0.013*		0.001*	

*: statistically significant

*CH* corneal hysteresis, *CRF* corneal resistance factor, *ORA* ocular response analyzer, *CST* Corvis ST, *IOP* intraocular pressure

**Fig. 2:**
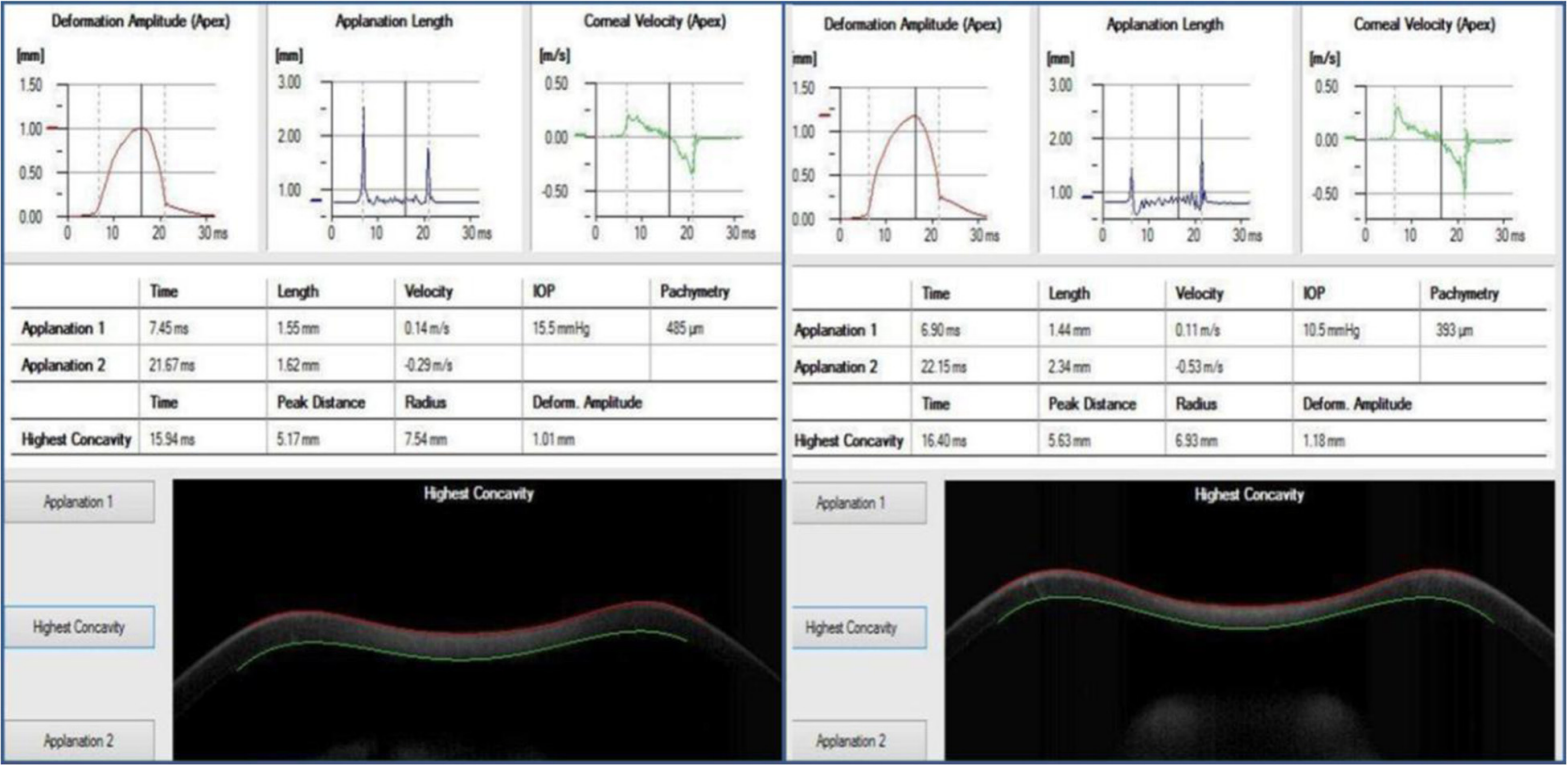
Showing CST corneal biomechanical changes in both groups with group I on the lest and group II on the right. Note the higher deflection amplitude in group II

The CST on the other hand also showed no significant difference between the preoperative and postoperative IOP in both groups with the postoperative IOP lower than the preoperative values (*p* = 0.2 and 0.382 respectively). The A2 time and HC time showed significant decrease from preoperative to postoperative values in both groups (*p* = 0.001), while the A1 time showed significant decrease in group II only (*p* = 0.001) and no significant change in group I (*p* = 0.69). It was also noted that the mean postoperative A1 time, HC time and A2 times were slower in group I (8.23 ± 0.37, 16.32 ± 1.10, 22.03 ± 1.11 respectively) than in group II (7.89 ± 0.44, 14.40 ± 1.27, 20.28 ± 1.87 respectively) denoting slower movements of the cornea in respect to the air puff (i.e. stiffer cornea). The A1, A2 lengths and HC peak distances showed also significant postoperative decrease in both group I and II (p ˂ 0.05). The HC radius of curvature significantly decreased in both group I and II postoperatively (*p* = 0.002 and 0.015 respectively). The mean postoperative HC radius of curvature in groups I and II were (6.91 ± 1.25 and 7 ± 1.06 respectively) and this was statistically insignificant (*p* = 0.389). However, the deflection amplitude increased significantly in both groups (*p* = 0.013 and 0.001 respectively) (Fig. 2).

The percentage of change of CH and CRF were found to be significantly lower in group II than in group I (*p* = 0.001). The percentage of change of the IOP was not significantly different in both groups pre and postoperatively using both the ORA and the CST (*p* = 0.23 and 0.312 respectively). The percent of change of A1 time, HC time and A2 time was significantly lower in group I than group II (*p* = 0.001, 0.004 and 0.001 respectively). The mean percentage of change in A1 length was not significant between both groups while the mean percentage of change in A2 length was significant (*p* = 0.152 and 0.044 respectively). The mean percentage of change in HC radius of curvature was significantly higher in group I (*p* = 0.001) while the mean percentage of change of HC peak distance and deflection amplitude were significantly higher in group II (*p* = 0.001). It was also noted that the mean change of the deflection amplitude was nearly five times higher in group II than group I denoting greater mobility of the cornea in this group (Table 4).

**Table 4:** The percentage of preoperative to postoperative changes in corneal biomechanics between both groups

%ge of change		CH	CRF	IOPcc ORA	IOP CST	A1 time	A2 time	A1 length	A2 length	HC time	HC R	HC P D	DA
Group I	Mean ± SD	- 17.1 ± 5.26	−17.36 ± 7.09	−2.36 ± 6.13	−5.4 ± 9.32	−2.03 ± 1.22	−6.73 ± 3.90	−9.60 ± 2.93	−7.55 ± 6.57	−11.28 ± 4.03	−13.6 ± 5.27	15.9 ± 6.8	5.13 ± 1.74
	range	−25.2to −6.8	−34.3 to-6.3	−12.1 to 14.6	−14.9 to 7.1	−3.7 to 0.0	−15.5 to-0.9	−17.5 to-5.6	−23.5 to 4.4	−21.2 to −3.0	−24.5 to −5.9	3.2 to 30.3	3.6 to 12.0
Group II	Mean ± SD	−27.04 ± 9.39	−33.30 ± 12.45	−6.59 ± 4.99	10.28 ± 7.28	−6.12 ± 1.49	−13.48 ± 5.17	−8.09 ± 3.29	−5.00 ± 3.24	−18.94 ± 5.27	−9.00 ± 4.22	28.99 ± 12.15	24.38 ± 7.97
	range	−54.3 to-14.9	−52.1 to −8.5	−16.9 to 1.8	−16.2 to 3.0	−8.6 to −3.3	−21.8 to −4.2	−14.0 to −3.4	−11.8 to 3.3	−26.7 to-7.1	−18.4 to-2.8	−2.6 to 50.3	9.3 to41.3
*P* value		0.001*	0.001*	0.23	0.312	0.001^*^	0.001^*^	0.152	0.044^*^	0.004*	0.001*	0.001*	0.001*

*: statistically significant

*CH* corneal hysteresis, *CRF* corneal resistance factor, *ORA* ocular response analyzer, *CST* Corvis ST, *IOP* intraocular pressure

## Discussion

The cornea is a very complex anisotropic tissue. The strength of the central cornea depends on interlamellar proteoglycan bonding whereas anterior and peripheral strength depend on branching and interlacing of lamellae. The anterior stroma is approximately 25 % stiffer than its posterior counterpart [17–20].

Several refractive procedures tend to alter the optical as well as the anatomical configurations of the cornea. New refractive procedures need to be fully investigated not only from the visual point of view but also from the safety point as well. Corneal biomechanics describes the response of corneal tissue to forces applied to them and entails interactions between the externally applied force, the intrinsic properties of the cornea as well as the IOP. Softer tissues tend to stretch or deform greater than stiffer tissues. The complexity of assessment of the in vivo corneal biomechanics is affected by several factors, thus hindering the use of simple or single factors as predictive biomechanical tools. Several investigators developed different machines in an attempt to assess corneal biomechanics including the ORA [6, 7], CST, Dynamic corneal imaging (DCI), Dynamic Rasterstereographic Corneal Topography (d.RCT), Optical Coherence Tomography Elastography, Corneal Transient Elastography (CTE) and Quantitative Ultrasound Spectroscopy (QUSi). However, only the ORA and the CST gained market popularity [9, 10].

Several studies have shown that myopic LASIK causes a general reduction in the ORA parameters namely; CH and CRF [10–21]. In our study, the CH and the CRF were significantly reduced in both groups postoperatively. However Kirwan et al. [24] and Medeiros et al. [25] stated that there was a non-significant reduction in CH after LASIK especially with thin flaps denoting that thinner flaps had no role on decreasing corneal biomechanics.

Both groups post-operatively had no significant change as regard the IOPcc and significant change as regard the IOP by the CST. However some studies [21–23] found a significant decrease of IOPcc after LASIK. This finding indicates that the ORA device does not completely compensate for the biomechanical properties of the cornea when measuring IOP. Also, IOP in all forms especially non-contact is largely dependent on corneal thickness. Table 2 shows that there is no statistically significant difference between the two groups either pre- or post-operatively.

Frings et al. [26] used the CST to measure corneal biomechanics pre and post LASIK and demonstrated a significantly shortening of A lengths as well as a higher deflection amplitudes and differences in the radius of curvature in the post-LASIK group. In addition, the HC was shorter, whereas the peak distance became longer post LASIK. These findings are consistent with our results as there was a significant reduction in A2 time, HC times, A1 length and HC corneal radii in both groups postoperatively. However, there was significant increase in the postoperative HC peak distance and deflection amplitudes in both groups.

When comparing the mean percentage of change of corneal biomechanics between both groups, we found significant difference regarding the CH and CRF with greater reduction of the corneal biomechanics in the LASIK group. This was also reported by other studies [4, 27].

Also in our study, the LASIK group showed a significant reduction regarding the mean percentage of change of almost all the biomechanical data except for the IOP by the CST and the A1 length. Of greater interest was the nearly fivefold increase in the mean percentage of change of the deformation amplitude in the LASIK group denoting much lower biomechanical change. These differences in the biomechanical behavior between both groups in our study can be explained by three factors. First, the microkeratome creates a meniscus flap extending deeper in the peripheral stronger corneal layers thus severing more biomechanically vital collagen bundles. Second, is the differential healing pattern perhaps with more inflammation with the femto SMILE group resulting in stronger fibrotic scarring as stated in previous studies [12, 14]. Third, was the difference of the flap to cap diameters as flaps tended to be bigger than the transition zones in the LASIK group (more than 8.5 mm) while the usual cap diameter in the femto SMILE cases was usually less than 8 mm thus also salvaging cutting the stronger peripheral collagen bundles.

The limitation of the current study is the small sample number of each procedure. Also the study did not involve a follow up period. The study was based on the assumption that most of the biomechanical changes after refractive procedures occur within 1 week of surgery as stated in previous studies [28, 29]. Also, one of the factors affecting the corneal biomechanics is the state of corneal dryness. This further adds to the difficulties encountered as some patients seeking refractive surgeries are already contact lenses intolerant with significant amount of dry eye [30].

There were several previous attempts to evaluate the corneal biomechanical changes after refractive surgeries. Sefat et al. [31] evaluated the changes in human corneas after femtosecond laser-assisted LASIK and SMILE using Corvis ST. Corneal biomechanical parameters measured preoperatively with Corvis ST showed significant differences postoperatively in total and in both groups. In subgroup analysis with homogenous groups, FS-LASIK showed no significant changes in biomechanical data measured with Corvis ST compared with SMILE. Also, Mastropasqua et al. [32] evaluated corneal biomechanical properties modification after SMILE using Scheimpflug-based noncontact tonometer. No significant modifications in biomechanical properties were observed after SMILE so this procedure could induce only minimal transient alterations of corneal biomechanics. While Shen et al. [33] evaluated changes in corneal deformation parameters after lenticule creation and extraction during SMILE procedure. There was a significant change in corneal deformation parameters following SMILE procedure. They suggested that the changes may be caused predominantly by stromal lenticule extraction, while lenticule creation with femtosecond laser may not have an obvious effect on corneal deformation properties. The current study combines two different tools to compare the corneal mechanical stability of the novel SMILE procedure to the standard LASIK procedure. To our knowledge this is one of the first studies that measures the corneal biomechanics using two different machines in the same study and on the same patients thus adding to the strength of the comparison and hence the strength of the study.

## Conclusions

In conclusion, we found that LASIK and SMILE substantially decreased the corneal biomechanical properties as measured by the ORA and the CST. Further studies of the morphology of the ORA signals and the CST data are necessary to fully clarify the biomechanical effect of the femto SMILE technique. Also the use of these tools is yet to demonstrate the biomechanical effect of femto SMILE if used at variable corneal depths or at variable optical zones (ongoing research).

## Abbreviations

A1 length, cord length of applanated cornea during A1; A1 time, is the time from starting until first corneal inward applanation; A2 length, cord length of applanated cornea during A2; A2 time, time from starting until second corneal outward applanation; CH, corneal hysteresis; CRF, corneal resistance factor; Deformation amplitude (DA), maximum inward movement of corneal apex at point of HC; HC peak distance (HCPD), distance between corneal peaks at point of highest concavity (HC); HC radius, radius of curvature of the corneal concavity at point of HC; HC time, time from starting until highest corneal concavity is reached; IOP CST, is the intraocular measurement based on A1 time; IOPcc, intraocular pressure corneal thickness compensated
